# Supplementary material for: Alisma Orientalis Extract Ameliorates Hepatic Iron Deregulation in MAFLD Mice via FXR-Mediated Gene Repression
Source: Nutrients. 2024 Jul 15;16(14):2272. doi: 10.3390/nu16142272 (PMC11279993; doi:10.3390/nu16142272)
Supplement: Supplementary file 1 [file nutrients-16-02272-s001.zip › nutrients-3052474-supplementary.pdf]

Alisma Orientalis Extract Ameliorates Hepatic Iron Deregulation in MAFLD Mice via FXR-Mediated Gene Repression

Yanlin Li [1,2], Ke Zhang [1,2], Yue Feng [1,2], Lei Wu [1,2], Yimin Jia [1,2] and Ruqian Zhao [1,2,3,*]

[1] MOE Joint International Research Laboratory of Animal Health & Food Safety, Nanjing Agricultural University, Nanjing 210095, China
[2] Key Laboratory of Animal Physiology & Biochemistry, College of Veterinary Medicine, Nanjing Agricultural University, Nanjing 210095, China
[3] National Key Laboratory of Meat Quality Control and Cultured Meat Development, Nanjing 210095, China
* Correspondence: zhaoruqian@njau.edu.cn

Table S1. Experimental diets composition and nutrient content.

	CON		HF	
Energizing material	gm%	Kcal%	gm%	Kcal%
Protein	19.2	20	26.2	20
Carbohydrate	67.3	70	26.3	20
Fat	4.3	10	34.9	60
Total		100		100
	3.85		5.24	
Ingredient	gm	Kcal	gm	Kcal
Casein, 80 Mesh	200	800	200	800
L-cystine	3	12	3	12
Corn starch	315	1260	0	0
Maltodextrin 10	35	140	125	500
Sucrose	350	1400	68.8	275.2
Cellulose	50	0	50	0
Soybean Oil	25	255	25	255
Lard	20	180	245	2205
Mineral mix S10026	10	0	10	0
Dicalcium Phosphate	13	0	13	0
Calcium Carbonate	5.5	0	5.5	0
Potassium Citrate,1 H_20	16.5	0	16.5	0
Vitamin Mix V10001	10	40	10	40
Choline Bitartrate	2	0	2	0
Pigment	0.05	0	0.05	0
Total	1055.05	4057	773.85	4057

Table S2. Nucleotide sequences of primers.

Target genes	Primer sequences (5′to 3′)	Used for
PPAR γ	F: CTTGCAGTGGGGATGTCTCA<br>R: CCTCGCCTTTGCTTTGGT	Real-time PCR
CD36	F: TTGATGTGCAAAATCCACAGG<br>R: TGTGTTGTCCTCAGCGTCCT	Real-time PCR
ACC1	F: GGAGATGTACGCTGACCGAG<br>R: TACCCGACGCATGGTTTTCA	Real-time PCR
FASN	F: GGCCCCTCTGTTAATTGGCT<br>R: GGATCTCAGGGTTGGGGTTG	Real-time PCR
SCD1	F: CCTCCGGAAATGAACGAGAGA<br>R: ATCCCGAAGAGGCAGGTGTA	Real-time PCR
TFR1	F: TCGGAGAAACTGGACAGCAC<br>R: AGGTGAGGTCTCCCTTCGTC	Real-time PCR
TFR2	F: GGTCTATTCCAGAGAGCGCA<br>R: CGACGTAGCCCAGTAGGAAG	Real-time PCR
ZIP14	F: AGAAGGTCATTGTGGGCTCG<br>R: AGTGAAGGAAGCACCGATGG	Real-time PCR
DMT1	F: AGCTGTCATCATGCCACACA<br>R: AGACTTCAACCACCTGCTCG	Real-time PCR
FTL	F: ATTTCGACCGCGATGATGTG<br>R: CATGGCGTCTGGGGTTTTAC	Real-time PCR
FTH	F: GCCATCAACCGCCAGATCAA<br>R: AAGATTCGGCCACCTCGTTG	Real-time PCR

FPN	F: GAGATCACAACCGCCAGAGA<br>R: CACATCCGATCTCCCCAAGT	Real-time PCR
HAMP	F: CTTTGCACGGGGAAGAAAGC<br>R: TGCAGATGGGGAAGTTGGTG	Real-time PCR
GST	F: CCATTGTACCACCTGGCCTT<br>R: TCGGCCATGCTTCCAATCTT	Real-time PCR
CAT	F: CACTGACGAGATGGCACACT<br>R: TGTGGAGAATCGAACGGCAA	Real-time PCR
SOD	F: AGGAGAGTTGCTGGAGGCTA<br>R: TAGTAAGCGTGCTCCCACAC	Real-time PCR
NQO1	F: CATTGCAGTGGTTTGGGGTG<br>R: TCTGGAAAGGACCGTTGTCG	Real-time PCR
HO-1	F: GGAAATCATCCCTTGCACGC<br>R: TGTTTGAACTTGGTGGGGCT	Real-time PCR
GCLC	F: CACAAGGACGTGCTCAAGTG<br>R: GTCGGATGGTTGGGGTTTGT	Real-time PCR
KEAP	GATGGGCAGGACCAGTTGAA<br>CCGAGGACGTAGATCTTGCC	Real-time PCR
PPIA	F: GACTGAGTGGTTGGATGG<br>R: TGATCTTCTTGCTGGTCTT	Real-time PCR
SCD1 Fragment 1	F: CTTCAGAGACCTTGTCTAAAA<br>R: GGACTAAGGAATCAAAGGGCCT	ChIP PCR
SCD1 Fragment 2	F: CTTCATAGATCTGTGTCTGCCT<br>R: GGATTAAAGGCATGCGCCACCA	ChIP PCR

FPN Fragment 1	F: ACCTCCAGGGCCCTCAGTTGCC<br>R: AGGCTTCCTGTAACAGTGGAT	ChIP PCR

Table S3. Antibodies used in this study.

Antibodies	Catalog Number	Source	Dilution Ratio
Tubulin-α	BS1699	Bioworld, China	1:5000
β-actin	AC026	ABclonal, China	1:1,00000
FASN	3189	Cell Signaling Technology, USA	1:1,000
PPARα	15540-I-AP	Proteintech, USA	1:1,000
SCD1	2438S	Cell Signaling Technology, USA	1:1,000
PPAR γ	WL01800	Wanleibio WL01800	1:500
CD36	BS7861	Bioworld, China	1:1,000
GR	ab183127	ABclonal, China	1:1,000
LXRα	A2141	ABclonal, China	1:1,000
FXR	A8320	ABclonal, China	1:1,000
RXR	A15242	ABclonal, China	1:1,000
TFR1	ab84036	Abcam, USA	1:1,000
TFR2	ab80194	Abcam, USA	1:1000
FPN	DF13561	Affinity, China	1:1000
